# An effective biomedical document classification scheme in support of biocuration: addressing class imbalance

**DOI:** 10.1093/database/baz045

**Published:** 2019-04-25

**Authors:** Xiangying Jiang, Martin Ringwald, Judith A Blake, Cecilia Arighi, Gongbo Zhang, Hagit Shatkay

**Affiliations:** 1 Department of Computer and Information Sciences, University of Delaware, Newark, DE, USA; 2 The Jackson Laboratory, 600 Main St., Bar Harbor, ME, USA; 3 Center of Bioinformatics and Computational Biology, Delaware Biotechnology Institute, Newark, DE, USA

## Abstract

Published literature is an important source of knowledge supporting biomedical research. Given the large and increasing number of publications, automated document classification plays an important role in biomedical research. Effective biomedical document classifiers are especially needed for bio-databases, in which the information stems from many thousands of biomedical publications that curators must read in detail and annotate. In addition, biomedical document classification often amounts to identifying a small subset of relevant publications within a much larger collection of available documents. As such, addressing class imbalance is essential to a practical classifier. We present here an effective classification scheme for automatically identifying papers among a large pool of biomedical publications that contain information relevant to a specific topic, which the curators are interested in annotating. The proposed scheme is based on a meta-classification framework using cluster-based under-sampling combined with named-entity recognition and statistical feature selection strategies. We examined the performance of our method over a large imbalanced data set that was originally manually curated by the Jackson Laboratory’s Gene Expression Database (GXD). The set consists of more than 90 000 PubMed abstracts, of which about 13 000 documents are labeled as relevant to GXD while the others are not relevant. Our results, 0.72 precision, 0.80 recall and 0.75 f-measure, demonstrate that our proposed classification scheme effectively categorizes such a large data set in the face of data imbalance.

## Introduction

The published literature is an important source of biomedical knowledge, as much information is conveyed in the form of publications. However, the large and increasing volume of published articles makes it impractical for researchers to quickly find all relevant documents related to their topic of interest. One way to address this challenge is through automated document classification, that is, identifying publications relevant to a specific topic within a large collection of articles. As such, automated biomedical document classification has attracted much interest (1–6). It is especially needed for the bio-databases curation workflow, as much information is manually curated within such databases (7), e.g. the Mouse Genome Informatics (MGI) database (8). Curators scan through a large number of publications to select those that contain relevant information—in a process known as *triage*. Automated biomedical document classification can provide an efficient and effective mean for supporting the time-consuming manual triage process.

### Background

The MGI database forms the most extensive international resource for the laboratory mouse. It provides integrated genetic, genomic and biological data for facilitating the study of human health and disease. Several databases contribute to MGI, such as the Mouse Genome Database (9), the Gene Expression Database (GXD) (10) and the Mouse Tumor Biology database (11). Here we focus on the GXD, which is a comprehensive, easily searchable and freely available database concerning expression information in the developing mouse. GXD collects and integrates RNA and protein expression information from RNA *in situ* hybridization, immunohistochemistry, *in situ* reporter (knock-in), RT-PCR, northern blot and western blot experiments. Expression data from wild-type and mutant mice are captured, with a primary emphasis on endogenous gene expression during development. Knock-in reporter studies are also included because they usually reflect the endogenous expression pattern of the targeted gene. Publications that report on endogenous gene expression during development and in postnatal stages are included. Excluded from the collection are studies reporting ectopic gene expression via the use of transgenes, experiments studying the effects of treatments or other external/environmental factors or papers that report only on postnatal gene expression.

Notably, much of the detailed information provided by GXD is manually curated from the literature. GXD curators scan about 140 journals surveyed by MGI to identify (triage) those publications that meet the above criteria. Once the publications are selected based on assessing the full-text of the article, the curators annotate the genes and the ages analyzed, as well as the types of expression assays used. These annotations and bibliographic metadata pertaining to the corresponding publications from PubMed (12) are used to create a searchable index of published experiments concerning endogenous gene expression during mouse development. This index supports quick access to publications discussing specific types of expression data. It thus helps expedite prioritizing publications for further detailed annotation of expression results within GXD. The comprehensive up-to-date index includes nearly 16 000 genes and more than 26 000 references containing data about endogenous gene expression. As mentioned before, the majority of GXD records that contain gene expression information are manually annotated. Moreover, the large and increasing number of biomedical documents being published each month makes keeping track of the latest publications and information access an onerous task. As such, it is important to build an effective biomedical document classifier for automating and accelerating the triage process in GXD to partition publications identified by MGI into those that are relevant to GXD and those that are not.

### Related Work

Much work over the past two decades aimed to address biomedical document classification. Most of the proposed methods are trained and tested over balanced data sets, in which all classes are similar in size (13–16). However, biomedical data sets are typically highly imbalanced, where relatively few publications within a large volume of literature are actually relevant to any specific topic of interest (17). Therefore, addressing class imbalance is essential for building practical biomedical document classifiers.

Several methods have been proposed for document classification under imbalance. Sampling strategies have been widely used—either removing data from the majority class (*under-sampling*) or adding duplicated/artificially generated data to the minority class (*over-sampling*) (18–21). For instance, Rahman and Davis (19) proposed cluster-based under-sampling to address class imbalance for categorizing cardiovascular records into high risk and low risk. Schneider *et al.* (21) employed random over-sampling to balance the data set for training a classifier identifying articles that describe protein–protein interactions. However, the above two classifiers were only applied over relatively small training/test data sets containing several hundreds to a few thousands documents. As such, these classifiers have not been shown applicable to a large-scale triage task, such as the one addressed in the context of GXD. Moreover, the method proposed by Schneider *et al.* used Medical Subject Headings (MeSH) terms (22) as features for document representation; these terms are assigned to articles by the U.S. National Library of Medicine (23) only several months after publication. As GXD directly curates new articles as soon as they are available, a classification system relying on MeSH terms annotations is not an effective route to pursue.

Larger-scale experiments were reported by Almeida *et al.* (5), who compared the performance of various classifiers (i.e. Naïve Bayes and Support Vector Machine) combined with different sampling strategies for handling class imbalance in triage for the mycoCLAP database (24), which comprises articles discussing fungal proteins. While the *recall* is quite high (~0.8), their reported *precision* and *f-measure* are low (<0.5). On a large data set of GXD’s magnitude, low precision typically implies much additional effort for re-checking the many false positives, deeming such a triage system ineffective for large-scale classification in the face of data imbalance.

In addition to sampling strategies, one-class learning (25, 26) has also been broadly applied to imbalanced document classification, and typically shown useful when applied to extremely imbalanced data sets where more than 90% of the data falls into one class (26, 27). In contrast, our data set is characterized by a lower imbalance ratio (i.e. ratio between the number of irrelevant documents to that of relevant documents) of ~ 6:1 (see details in the next section). As such, one-class learning is not applicable here.

Several automated document classification systems have been developed specifically to be incorporated into the triage process in bio-databases such as WormBase (28) or MGI. The work most related to ours is by Fang *et al.* (2), aiming to address triage tasks within the context of WormBase, FlyBase (29) and MGI. It employs an ensemble of Support Vector Machines (SVMs) classifiers along with random under-sampling to address class imbalance. While the classification scheme has been successfully applied over small data sets (<1300 documents), when applied to the large imbalanced data set we consider here, the scheme does not perform as well (<0.7 precision, recall and f-measure) thus leaving room for improvement (see analysis in the Experiments and Results section). We also note that the proposed system was trained and tested using full-text of publications, which are typically in PDF format; gathering such documents on a large scale and correctly extracting text contents from them is challenging (30). As relying on the readily available titles-and-abstracts has been shown useful for triage (31, 32), we develop here an effective classification system relying on title-and-abstract toward supporting the GXD triage process.

To summarize, many of the existing methods have only been applied to relatively small data sets, while others have not shown good performance over imbalanced classes. As such, these methods have not been shown effective for categorizing imbalanced data sets of the magnitude that curation efforts, such as GXD’s, face in practice.

In our own preliminary work (13), we presented an effective—yet relatively simple—classification scheme using readily available tools, while employing several of our statistical feature selection strategies, for identifying publications relevant to GXD among a large set of MGI documents. Our proposed method attained high performance (>0.9 on all performance measures) when trained and tested over a large balanced data set of curated GXD publications. When applied to a large but imbalanced data set, the recall dropped to 0.88 while precision dropped to 0.43 (f-measure 0.58). As mentioned above such low precision deems the classifier ineffective on a large imbalanced data set.

In this work, we train and test a binary document classifier using a large, imbalanced well-curated data set for supporting triage in GXD. The data set is a collection of abstracts from publications labeled by MGI throughout the years 2004–2014. Specifically, we propose a classification framework to partition the set of publications examined by MGI into those that are relevant to GXD vs those that are not. We present a modified *meta-classification* scheme (33) using a cluster-based under-sampling method, combined with document representation models employing statistical feature selection and named-entity recognition (NER) (34). Our reported performance on a set of over 90 000 documents is 0.72 precision, 0.80 recall, 0.75 f-measure and 0.71 Matthews correlation coefficient (MCC) (35), which significantly exceeds the reported performance of an earlier classifier aiming to address similar triage tasks in the face of data imbalance (2). This level of performance demonstrates that our method effectively addresses class imbalance and is applicable to a realistic large-scale triage task.

## Methods

### Data

We train and test our classifier over a large and well-curated imbalanced data set, namely, a collection of documents from the periods 2004–2014 selected by MGI. All the documents are downloaded from PubMed. In this study, we focus on the task of binary document classification, that is, identifying publications that are relevant to GXD within the MGI database.

As mentioned in the Introduction, titles and abstracts of scientific publications are readily available and proven sufficient for biomedical document classification (31, 32). We thus use the data set comprising 91 860 abstracts (harvested from PubMed) for training and testing the proposed classification scheme. Among these documents, 12 966 are labeled as relevant to GXD and comprise the *positive* (relevant) set, while the remaining 78 894 are labeled as irrelevant and comprise the *negative* (irrelevant) set. The imbalance ratio, as noted earlier, is ~6:1.

### Classification framework

Using off-the-shelf packages to train and test classifiers over an imbalanced data set typically leads to poor performance, because the learned classifiers are biased toward the majority class—the irrelevant class in our case. Additionally, employing common methods for addressing the imbalance, such as random under-sampling, often leads to discarding potentially useful data (5, 18). To address such challenges in the face of large-scale imbalanced data sets, we employ a meta-classification scheme, which combines the results obtained from multiple simple classifiers into a single classification decision, along with a cluster-based under-sampling method over the majority class (the irrelevant class).

The meta-classification framework comprises two sub-tasks: first, a set of *K* simple classifiers, {$C_{1},C_{2}...,C_{K}$} referred to as the *base-classifiers*, are trained. To categorize a document, $d$, each base-classifier is applied and assigns a prediction score $C^{d}_{i} \,(i \in \{{1, 2,..., {K}\}})$, where $C^{d}_{i} = Pr \,(d \in the\, relevant\, class \boldsymbol|C_{i})$, which is the probability of the document $d$ to be identified as relevant by the *i*th base-classifier. The results from *K* base-classifiers are then used to re-represent the document as a $K$-dimensional vector $\langle{C^{d}_{1}, C^{d}_{2},..., C^{d}_{K}\rangle}$, consisting of the prediction score assigned by each base-classifier. This representation is used for training another classifier, referred to as the meta-classifier, which assigns the final class label to each document.

To train the base-classifiers, we first employ under-sampling over the irrelevant set to reduce the gap between the number of relevant articles and that of irrelevant ones. Notably, the irrelevant documents discuss a variety of distinct sub-areas (such as tumor biology and genomic mutations), where every sub-area forms its own cohesive subset. Therefore, each such irrelevant cohesive subset alone has the potential to be individually distinguished from the relevant class. However, simply employing the rudimentary and widely used random under-sampling splits the irrelevant class at random into heterogeneous subsets, each covering a multitude of topics. While each individual document in such a subset carries salient features that are likely to distinguish it from the documents in the relevant set (and other terms that are possibly similar to those appearing in relevant documents), these features are unlikely to be shared by the majority of documents in a topically heterogeneous subset. As such the heterogeneous subsets are not readily distinguishable from the relevant set of documents. To remedy that, here we employ a partitioning strategy—a variation on cluster-based under-sampling (19)—that aims to identify topically coherent clusters within the irrelevant documents. Each cluster corresponds to a subset of documents covering a cohesive sub-area, where each such irrelevant subset can be distinguished from the relevant class by training an appropriate base-classifier.

Specifically, we employ K-means clustering (36) to partition the irrelevant set into *K* clusters, using cosine distance as the similarity metric. As such, the large irrelevant set is divided into *K* subsets, each covering a distinct area or topic. We then train each of the *K* base-classifiers to distinguish one of these *K* subsets from the relevant set. We use Random Forest classifier (37), which has proven effective for high-dimensional data, as the base-classifier.

**Figure 1:**
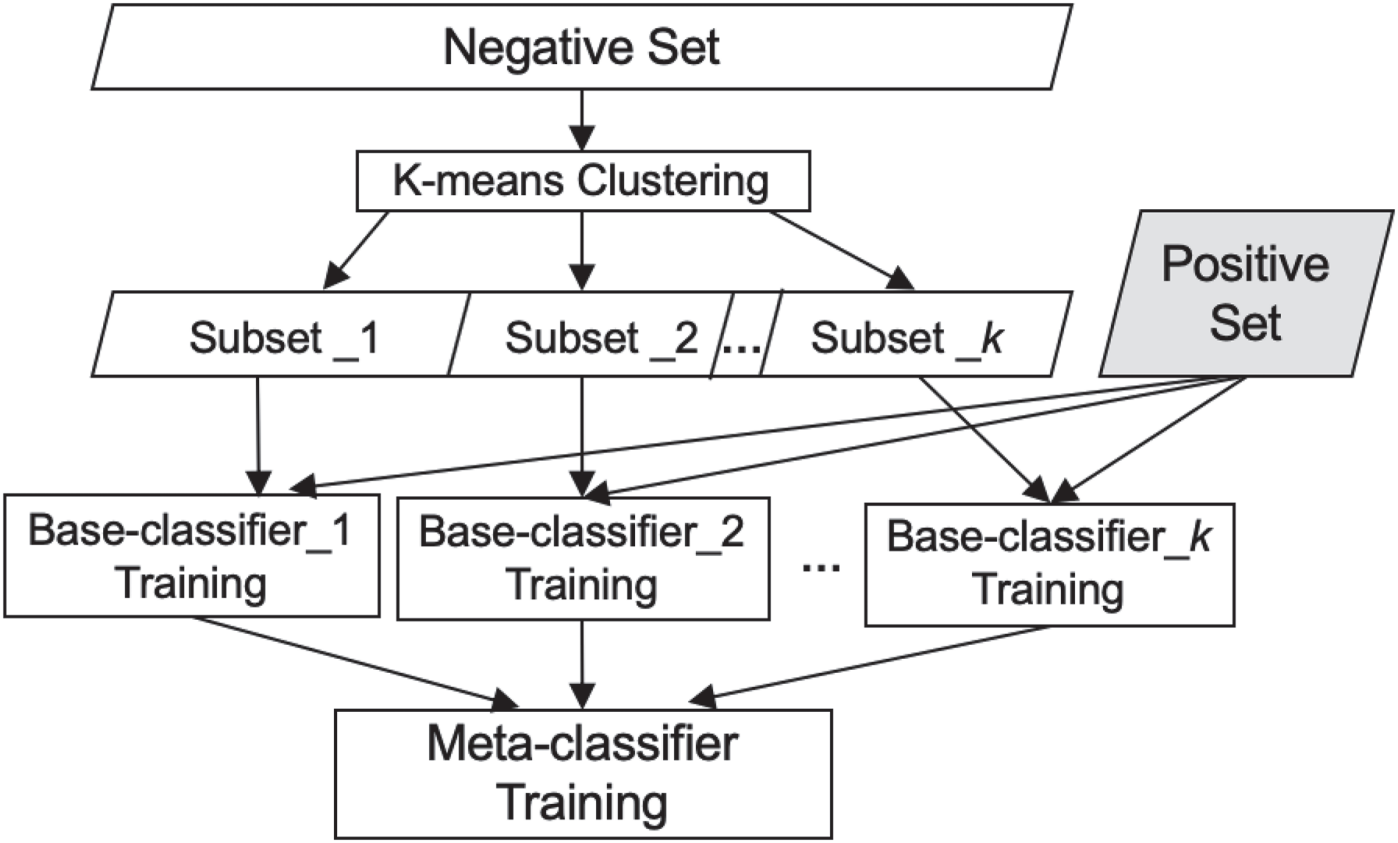
Our classification scheme, combining clustering and meta-classification. The irrelevant training set is partitioned into K subsets via K-means clustering. Each of the K base-classifiers is trained using one of these K irrelevant subsets along with the relevant training set.

To choose the meta-classifier, we ran experiments utilizing several widely employed classifiers, namely Naïve Bayes, Random Forest and SVMs (38), using each of them as the meta-classifier*.* As SVM performed best (see comparison in the Experiments and Results section) and has been shown effective by others as well (39), we use it as the meta-classifier. Figure 1 summarizes our classification scheme.

### Document representation

Notably, *K*-means clustering is employed over the whole irrelevant set, while the base-classifiers learning is conducted over each of the sampled irrelevant subsets along with the same relevant set. As such, we employ different feature selection steps for document representation when conducting *K*-means clustering and when training/testing the base-classifiers, as discussed below.

Our initial document representation is based on the bag-of-words model, used in our earlier work (40, 41). The set of terms consists of both unigrams (single words) and bigrams (pairs of two consecutive words). Using a limited number of meaningful terms as features for document representation has been proven effective in our earlier work (40, 41). To reduce the number of features, we first annotate documents using two readily available biomedical NER tools, Pubtator (42–44) and BeCAS (45). These NER tools allow identification of gene, enzyme, protein and mutation concepts. We then substitute all gene and protein concepts (e.g. GRP and S1P) by the generic term *PRGE*, while specific mentions of enzymes (e.g. IKK and PKC) or of mutation concepts (e.g. M146V) are similarly replaced by the generic terms *ENZI* or *MUTN,* respectively. We remove standard stop words, single letters, rare terms (appearing in fewer than 50 documents in the data set) and frequent terms (appearing within over 60% of the data set).

We also employ the Z-score test (40, 46), which we used before, to select features whose probability to occur in the relevant set is statistically significantly different from that to occur in the irrelevant class. Let *t* be a term, *D_rel_* denote the relevant set, while *D_irrl_* denote the irrelevant class. The probability of a term *t* to occur within the relevant set, $Pr
\,(t\boldsymbol|D_{rel}\,)$, is calculated as

$$Pr (t \boldsymbol|D_{rel}) = \frac {\#\, of\, documents\, in\, D_{rel}\, that\, have\, term\, t} {total\, 
\#\, of\, documents\, in\, D_{rel}} .$$

Similarly, the probability of a term *t* to appear in the irrelevant set, $Pr\, (t\boldsymbol|D_{irrl})$, is estimated as

$$Pr
(t \boldsymbol|D_{irrl}) = \frac {\#\, of\, documents\, in\, D_{irrl}\, that\, have\, term\, t} {total\, \#\, of\, documents\, in\, D_{irrl}}.$$

We calculate the probabilities $Pr\,(t\boldsymbol|D_{rel}\,)$ and $Pr\, (t\boldsymbol|D_{irrl})$ for each term *t*. To determine the significance of the difference between these two probabilities, the Z-score statistic is employed. The higher the absolute value of Z-score, the more statistically significant the difference between $Pr\,(t\boldsymbol|D_{rel}\,)$ and $Pr\, (t\boldsymbol|D_{irrl})$. Therefore, we consider a term *t* to be distinguishing with respect to our classification task if the Z-score of the term *t* is higher than a predetermined threshold, which is set to 1.96 here. We refer to each such selected term as a *distinguishing term*. Notably, the above feature selection steps are applied only to the training set. In our experiments, the number of features selected to represent documents for *K*-means clustering using this process is ~15 000.

As we apply *K*-means to the irrelevant set, where each cluster serves as a sampled irrelevant subset, we first represent each irrelevant document *d* as a simple *m*-dimensional binary vector of the form $V^{d} = \langle{V^{d}_{1}, V^{d}_{2},..., V^{d}_{m}\rangle}$, where *m* is the number of features selected, and $V^{d}_{j} = {1}$ if the *j*^th^ distinguishing term appears in document *d*, 0 otherwise.

After clustering the irrelevant documents, represented as *m*-dimensional vectors, into *K* subsets, we develop *K* base-classifiers to distinguish between each irrelevant subset and the relevant set.

**Figure 2:**
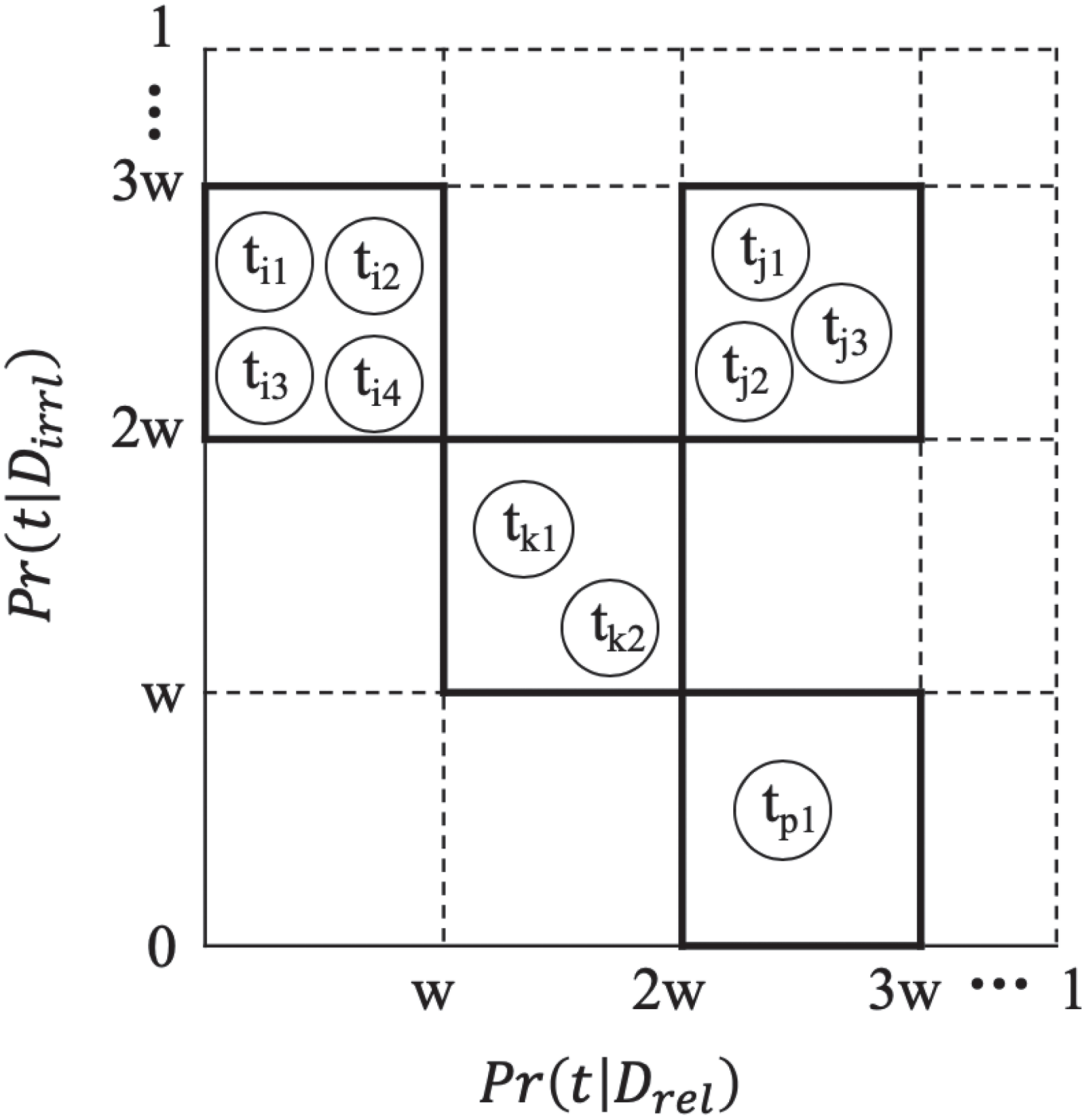
Feature Binning. To bin a set of terms, we first partition the continuous probability interval [0,1] into equal-spaced sub-intervals, each of width w. Two terms ${\mathrm{t}}_{\mathrm{p}}$ and ${\mathrm{t}}_{\mathrm{q}}$ are grouped into one bin if and only if the probability $Pr ({\mathrm{t}}_{\mathrm{p}}|{\mathrm{D}}_{r\mathrm{el}})$ falls into the same probability sub-interval as $P\mathrm{r}({t}_q|{D}_{r\mathrm{el}})$, and $P\mathrm{r}({t}_p|{D}_{i\mathrm{rrl}})$falls into the same probability sub-interval as $P\mathrm{r}({t}_q|{D}_{i\mathrm{rrl}})$.

While the vector representation described above may capture some of the salient features characterizing a GXD-relevant document compared to all irrelevant documents, each base-classifier needs to distinguish between only one specific subset of the irrelevant documents and the relevant set. We thus employ the feature selection process over each subset of documents used to train each base-classifier. Notably, the number of distinguishing terms selected by the process described above for document representation when training/testing each base-classifier is still high (~8000 to ~13 000), while each sampled irrelevant subset and the original relevant class consist of a relatively small number of documents (<13 000 articles for each base-classifier learning). Given this limited number of documents, to improve classification performance, we further reduce the dimensionality of the document-vectors by employing a binning strategy. For binning a set of *M* terms, we start with *M* distinct bins, and initialize each bin to contain a single term. We partition the continuous probability interval [0,1] into equally spaced sub-intervals, each of width *w* (where *w* was experimentally determined. Here we report results in which $w=0.0001$, as this yielded the best performance). At each step we merge a pair of bins if and only if all the terms in both bins are similar in their probabilities to occur in relevant documents, as well as in the irrelevant ones. That is, two bins *p* and *q* are merged if and only if for every term *t_p_* in *p* and *t_q_* in *q*, the probability $Pr({t}_p|{D}_{rel})$ falls into the same probability sub-intervals as $Pr({t}_q|{D}_{rel})$, and $Pr({t}_p|{D}_{irrl})$ falls into the same probability sub-intervals as $Pr({t}_q|{D}_{irrl})$. The merging process terminates when no pair of bins meets the merging criterion. We refer to this feature reduction process as *feature binning*. Figure 2 illustrates the feature binning process. In our experiments, the number of bins obtained ranges from ~2800 to ~3600 when representing documents toward training/testing the *K* base-classifiers.

As we train and test one base-classifier, we represent each document *d* in either the corresponding sampled irrelevant subset, the relevant set or the test set as a simple binary vector of the form $G^{d} = \langle{G^{d}_{1}, G^{d}_{2},...,
G^{d}_{n}\rangle}$ where *n* denotes the number of bins generated as discussed above, and $G^{d}_{u} = {1} $ if any term in the *u^th^* bin appears in document *d*, 0 otherwise. Notably, the dimensionality of the vectors used for training/testing each base-classifier varies based on the number of bins obtained.

To train and test the meta-classifier, we apply each of the base-classifiers to every document in the complete data set as discussed in the Classification Framework section. For document *d*, the *i*th base-classifier, which is a Random Forest classifier as mentioned in the Document Representation section, assigns a prediction score $C^{d}_{i} $ to the document, where

$C^{d}_{i} = Pr\, (d \in the\, relevant\, class \boldsymbol|C_{i})$.

Specifically, in our case,

$$ C^{d}_{i}
= \frac {\#\, of\, decision\, trees\, that\, predict\, d\, as\, relevant}{total\, \#\, of\, trees}$$

After processing all instances, each document *d* is re-represented as a *K*-dimensional numerical vector.

$ C^d = \langle C^d_1 , C^d_2, ..., C^d_K \rangle  $ using the *K* prediction scores assigned by the *K* base-classifiers.

## Experiments and Results

### Experiments

To ensure the stability of the results, we performed stratified 5-fold cross validation in all experiments. In each stratified cross validation run, 80% of the relevant documents and 80% of the irrelevant ones were used for training the complete classification scheme while the remaining 20% of the data set were used for testing. As for the meta- and base-classifiers training, the data within the training set was further split at random into two subsets: 75% of the original training data (60% of the complete data set comprising 60% of relevant documents and 60% irrelevant ones) was used for training the base-classifiers; the remaining 25% of the original training data (20% of the complete data set) was used for the meta-classifier training.

The meta-classifier selection was done by comparing three different commonly used classifiers for performing the final meta-classification step, namely, Naïve Bayes, Random Forest [implemented using Weka (47)] and SVM [LIBSVM library implementation (48)]. As SVM showed the best performance, we used it as the meta-classifier of choice throughout the rest of the experiments (see the Document Representation section for details).

The number of clusters, *K*, was chosen by running multiple experiments, in which the number of clusters ranged from 5 to 7. We note that the motivation for partitioning the negative set into subsets lies in the need to balance the size of each negative subset with that of the positive set, where the original imbalance ratio is ~6:1. Setting the *K*-value in the ranges 5–7 is likely to result in clusters that indeed accommodate such size balance. As the classification process attained its highest performance when *K =* 5 (see the Results and analysis section), the number of clusters *K* is set to 5 throughout the rest of the experiments described below.

We ran the whole system (with the selected meta classifier set to SVM and the number of clusters *K* = 5) over the data set described in the previous section, validating that our proposed classification framework indeed effectively addresses the class imbalance inherent in the GXD triage task.

To assess whether our feature selection steps indeed identify meaningful features that improve classification, we ran additional sets of experiments, employing the same meta-classification scheme (with *K* = 5 clusters) while representing documents based on three different feature selection procedures. In the first, we used feature selection steps including removing standard stop words, single letters, rare terms as well as frequent terms and employing the Z-score test, without conducting NER and feature binning to identify distinguishing terms. In the second, we added the feature binning step. In the third set, we executed all feature selection steps including NER as discussed in the Document Representation section.

We compare the performance of our whole system both to a baseline that uses random under-sampling (mentioned in the Classification Framework section) and to an earlier method proposed for addressing a similar triage task under imbalance (2). For the former, we divided the irrelevant set at random into five equal subsets, and trained five base-classifiers to distinguish between the relevant set and each of the irrelevant subsets obtained through random sampling. For the latter, we reimplemented the classification scheme proposed by Fang *et al.* (2) (which is the work most related to ours, as discussed in the Related Work section) and compare the performance attained by their classification scheme to that of ours over the current large data set used here.

### Results and analysis

We report the results using standard measures widely employed for document classification evaluation, namely precision, recall and f-measure (49)*.* In addition, we also report the *MCC*, a metric commonly employed in the context of classification under imbalance. MCC is defined as:

$$ MCC = \frac{TP  \times   TN  -  FP  \times  FN}{\sqrt{(TP  +  FP)(TP  +  FN)(TN  +  FP)(TN  + FN)}}, $$

where *TP* denotes the number of true positives, *TN* represents the number of true negatives, *FP* denotes the number of false positives and *FN* represents the number of false negatives.

The *MCC* ranges between −1 to +1, where −1 indicates total disagreement, +1 indicates perfect agreement, while 0 corresponds to random class assignments.

Table 1 shows the results attained from the first group of experiments in which we vary the classifiers used for meta-classification. While using Naïve Bayes as the meta-classifier leads to the highest recall and Random Forest attains the highest precision, SVM significantly outperforms both in terms of f-measure and MCC (*P* ≪ 0.001, two sample *t*-test), striking a good balance between precision and recall. Notably, the MCC is a particularly useful measure for assessing classification performance under data imbalance (49, 50).

**Table 1:** Results attained when varying the meta-classifiers (where the number of clusters used, *K*, is set to 5). Standard deviation is shown in parentheses. The highest performance level along each metric is shown in boldface

Meta-classifier	Precision	Recall	F-measure	MCC
Naïve Bayes	0.603 (0.007)	**0.871 (0.005)**	0.713 (0.005)	0.672 (0.006)
Random Forest	**0.776 (0.004)**	0.694 (0.008)	0.733 (0.003)	0.693 (0.004)
SVM	0.719 (0.008)	0.791 (0.012)	**0.753 (0.004)**	**0.711 (0.004)**

**Table 2:** Results attained varying the number of clusters, *K*, where *K*-means clustering is used to partition the irrelevant set into cohesive clusters. Standard deviation is shown in parentheses. The highest performance level along each metric is shown in boldface

Number of clusters	Precision	Recall	F-measure	MCC
K = 5	0.719 (0.008)	**0.791 (0.012)**	**0.753 (0.004)**	**0.711 (0.004)**
K = 6	**0.750 (0.007)**	0.700 (0.001)	0.724 (0.003)	0.678 (0.004)
K = 7	0.732 (0.027)	0.737 (0.055)	0.733 (0.014)	0.687 (0.014)

Table 2 shows the results from the experiments where we vary the number of clusters, *K.* The results indicate that setting the number of clusters to 5 leads to the highest recall, f-measure and MCC (*P* ≪ 0.001, two sample *t*-test).

As the f-measure takes into account both precision and recall, while MCC (as noted above) is useful for assessing classification under imbalance, the results shown in Tables 1 and 2 together indicate that using SVM as the meta-classifier while setting the number of clusters to 5 is the most effective route to pursue.

Table 3 compares the performance of our proposed meta-classification scheme under different feature selection settings. Row 1 summarizes the performance when employing most of the feature selection steps (stop-word removal, single letters, rare and frequent term removal and applying the Z-score test) except for NER normalization or feature binning. The number of features selected for representing the documents in the base-classifier learning in this set of experiments ranges from ~8000 to ~13 000. Row 2 shows the results when feature binning is also applied. The number of features selected is in the range of ~2700 to ~3600. Row 3 shows the performance when all feature selection steps discussed in the Document Representation section are conducted. The number of selected features in this case varies from ~2900 to ~4000. The results show that using all proposed feature selection steps leads to the highest recall, f-measure as well as MCC (shown in Row 3). Most of the differences between the results shown in the third row and those shown in the top two rows are highly statistically significant (*P* ≪ 0.001, two sample *t*-test). The others are also statistically significant (*P* ≤ 0.01), and even the difference between *MCC* in the third row (0.711) and that in the second (0.702) is still statistically significant (*P* = 0.02). These results demonstrate that our feature selection indeed identifies a limited number of meaningful features for document representation, while improving classification performance.

**Table 3:** Classification results under different feature selection settings. `NER’ denotes the NER step and `BIN’ represents the feature binning step. A ‘+’ sign represents employing the respective selection step, while a ‘–’ denotes its exclusion. Standard deviation is shown in parentheses. The highest performance level along each metric is shown in boldface

Feature selection methods	Precision	Recall	F-measure	MCC
NER-, BIN-	0.721 (0.013)	0.678 (0.006)	0.699 (0.007)	0.652 (0.009)
NER-, BIN+	**0.752 (0.004)**	0.736 (0.010)	0.744 (0.006)	0.702 (0.007)
Our final classifier (NER+, BIN+)	0.719 (0.008)	**0.791 (0.012)**	**0.753 (0.004)**	**0.711 (0.004)**

**Figure 3:**
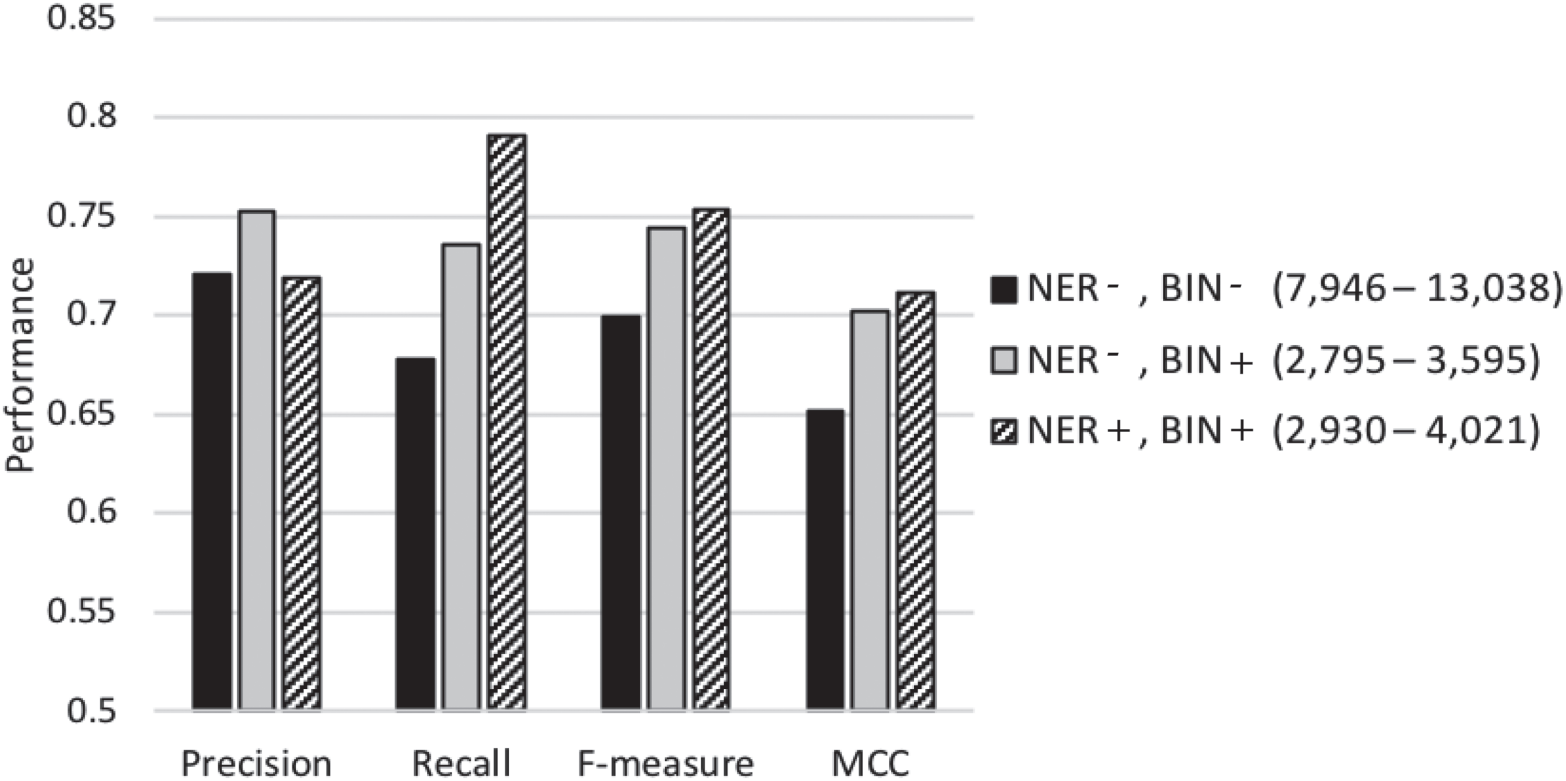
Performance of our classification scheme under different feature selection settings using 5-fold cross validation denotes the biochemical/genomic named entity recognition step and BIN represents the feature binning step. A ‘+’ sign represents employing the respective selection step, while a ‘−’ denotes its exclusion. The range of the number of features selected for the base-classifiers learning across the cross-validation runs is shown in parentheses.

Figure 3 graphically depicts the results shown in Table 3. As shown in the figure, when the average number of features selected for learning the base-classifiers is reduced from about 10 000 (columns shown in black) to about 3300 (columns shown in light gray) using feature binning, the precision, recall, f-measure and MCC all increase significantly. When NER strategy is also employed to identify and replace biomedically meaningful proper nouns or specific words by generic terms, the average number of selected features in the base-classifiers learning goes up to ~3600 (column shown in diagonal stripes). The recall, f-measure and MCC increase while the precision slightly decreases. Conducting NER leads to a slight increase in the number of features selected along with some improvement in classification performance. This demonstrates that meaningful and distinguishing generic terms are selected as features when employing limited NER for document representation. In summary, our results demonstrate that the NER strategy and feature binning are indeed beneficial as part of feature selection toward classification under imbalance.

Table 4 compares the performance attained by our whole system, both to that obtained via random under-sampling and to that of the ensemble SVM method proposed by Fang *et al.* (2) over our large data set. Our scheme (employing *K-*means with *K* = 5 over the irrelevant training set) attains the highest recall, as well as f-measure and MCC (see bottom row of the table), while random under-sampling shows higher precision*.* Specifically, using clustering to partition the large negative set leads to an improvement of about 10 percentage units in recall compared to random under-sampling, at the cost of only 3% in precision. The overall significant improvement demonstrates the value of using clustering to expose distinct and cohesive subsets within the large irrelevant set, thus allowing classifiers developed for each such subset to more effectively distinguish these subsets from the set of relevant documents.

**Table 4:** Performance attained by our classification scheme compared to that attained via random under-sampling and by the ensemble SVM classification method proposed by Fang *et al.* (2). Standard deviation is shown in parentheses. The highest performance level along each metric is shown in boldface

Method	Precision	Recall	F-measure	MCC
Random under-sampling	**0.741 (0.004)**	0.694 (0.006)	0.717 (0.004)	0.673 (0.005)
Ensemble SVM	0.692 (0.02)	0.642 (0.03)	0.662 (0.01)	0.613 (0.01)
Our final classifier (K-means, K = 5)	0.719 (0.008)	**0.791 (0.012)**	**0.753 (0.004)**	**0.711 (0.004)**

Moreover, our classification framework improves upon the ensemble SVM (second row in the table), which was proposed within a similar context, according to all performance measures. Most notably, both the f-measure and the MCC attained by our classifier are significantly higher—with high statistical significance (*P* ≪ 0.001, two sample *t*-test)—than those attained by the ensemble SVM, clearly demonstrating the effectiveness of our system for addressing triage in GXD.

## Conclusion and future work

We have presented a meta-classification scheme employing cluster-based under-sampling along with feature selection strategies for effectively identifying publications relevant to the mouse GXD over a realistically large and imbalanced data set. Our proposed classifier attains precision 0.72, recall 0.80, f-measure 0.75 and MCC 0.71. This level of performance is higher than any previously reported over large biomedical document data sets in the face of data imbalance. Our results show that the proposed meta-classification scheme along with employing *K*-means clustering over the irrelevant class is capable of addressing the class imbalance arising in the GXD triage task. Additionally, we note that our feature selection process, which includes statistical feature reduction along with named-entity tagging is useful for improving classification performance. Moreover, our classification scheme can be readily adapted to other triage tasks by incorporating appropriate annotation tags into the vocabulary based on the specific domain and by modifying specific classification parameters such as the number of base-classifiers used within the meta-classification.

As we demonstrated in our earlier work (13), image captions in biomedical publications, which form brief summaries of the images, contain significant and useful information for determining the topic discussed in the publications. As part of future work, we plan to integrate image captions into the classification scheme. We also intend to work on combining other sources of information, including associated sentences from the full text that discuss images, to further improve classification over large imbalanced data sets.
